# Suppression of microRNA-384 enhances autophagy of airway smooth muscle cells in asthmatic mouse

**DOI:** 10.18632/oncotarget.18913

**Published:** 2017-07-01

**Authors:** Zhe Cheng, Xi Wang, Lingling Dai, Liuqun Jia, Xiaogang Jing, Ying Liu, Huan Wang, Pengfei Li, Lin An, Meng Liu

**Affiliations:** ^1^ Department of Respiratory and Critical Care Medicine, Institute of Clinical Medicine, The First Affiliated Hospital of Zhengzhou University, Zhengzhou 450052, China

**Keywords:** asthma, ovalbumin (OVA), miR-384, beclin-1, autophagy, airway smooth muscle (ASM) cells

## Abstract

Injury to airway smooth muscle (ASM) cells hallmarks the pathological progression of asthma, a chronic inflammatory airway disease. MicroRNAs (miRNAs) play essential roles in the development of asthma as well as airway remodeling. Here we studied the involvement of miRNAs in the regulation of autophagic survival of ASM cells and airway disorder. We analyzed autophagy-associated factors LC3 and Beclin-1 by RT-qPCR and protein blotting in purified airway smooth muscle cells from ovalbumin (OVA)-induced asthmatic mice. The biological activity of miRNA targeting Beclin-1 was explored by bioinformatics method and confirmed in a dual luciferase reporter assay. Loss of function experiment was performed via transplantation of miRNA in OVA-induced asthmatic mice. We detected high autophagy levels in ASM cells, which appeared to result from augmentation of Beclin-1 protein, rather than Beclin-1 mRNA, suggesting presence of post-transcriptional control of Beclin-1. Next, miR-384 was figured out to be a Belcin-1-targeting miRNA, which significantly decreased after OVA treatment. Mechanistically, binding of miR-384 to 3’-UTR of Beclin-1 mRNA potently suppressed Beclin-1 protein translation in ASM cells, similar to previous finding in another cell type. In vivo, transplantation of miR-384 significantly attenuated Belcin-1 protein levels in ASM cells, resulting in reduced autophagy of ASM cells and attenuation of asthmatic features by OVA. Together, these data suggest that re-expression of miR-384 may reduce augmentation of Beclin-1-dependent autophagy of ASM cells, as a novel promising treatment for asthma.

## INTRODUCTION

Asthma is a common chronic disease characterized by bronchial inflammation, airway hyper-responsiveness, and airflow disorder and obstruction. Asthma affects nearly 30 million people in the United States and many more worldwide. As the most common chronic disease in childhood, the effective control and treatment of asthma appears to be essential for health protection of millions of children [1, 2].

Airway smooth muscle (ASM) cells are main effector cells of airway disorder, narrowing and obstruction [3]. Airway hyper-responsiveness, inflammation and remodeling are also partially attributable to airway smooth muscle, since ASM cells exhibit exaggerated responses to bronchoconstrictor stimuli under airway hyper-responsiveness, alter cell mass and deposition of extracellular matrix, as well as secrete inflammation-associated cytokines, chemokines and growth factors [3]. However, the effects of autophagic survival of ASM cells on asthma development remain unknown.

Autophagy is a biological choice for the cells to survive nutrient deprival or harsh environment through recycling degraded cellular compartments [4]. During autophagy, autophagosomes engulf degraded cellular compartments, leading to conjugation of cytosolic microtubule-associated protein 1A/1B-light chain 3 (LC3-I) to phosphatidylethanolamine LC3-phosphatidylethanolamine conjugate (LC3-II). The ratio of LC3-II to LC3-I levels is thus a representative for the autophagic activity to be used commonly [4–6]. Autophagy-associated protein 6 (Atg6, or Beclin-1) is an essential autophagy regulator that potently coordinates the initiation and progression of autophagy [7]. Interestingly, small non-coding RNAs, or microRNAs (miRNAs), were recently shown to control Beclin-1 levels in some type of cells. Specifically, miR-384 was a miRNA that was involved in the regulation of neural functions [8, 9], and targeted Beclin-1 to control macrophage autophagy in the development of atherosclerosis [10]. However, the role of this regulatory axis in ASM cells and asthma development has not been examined. Here, we addressed this question.

## RESULTS

### Enhanced ASM cell autophagy is detected in mouse lung after OVA treatment

**Figure 1:**
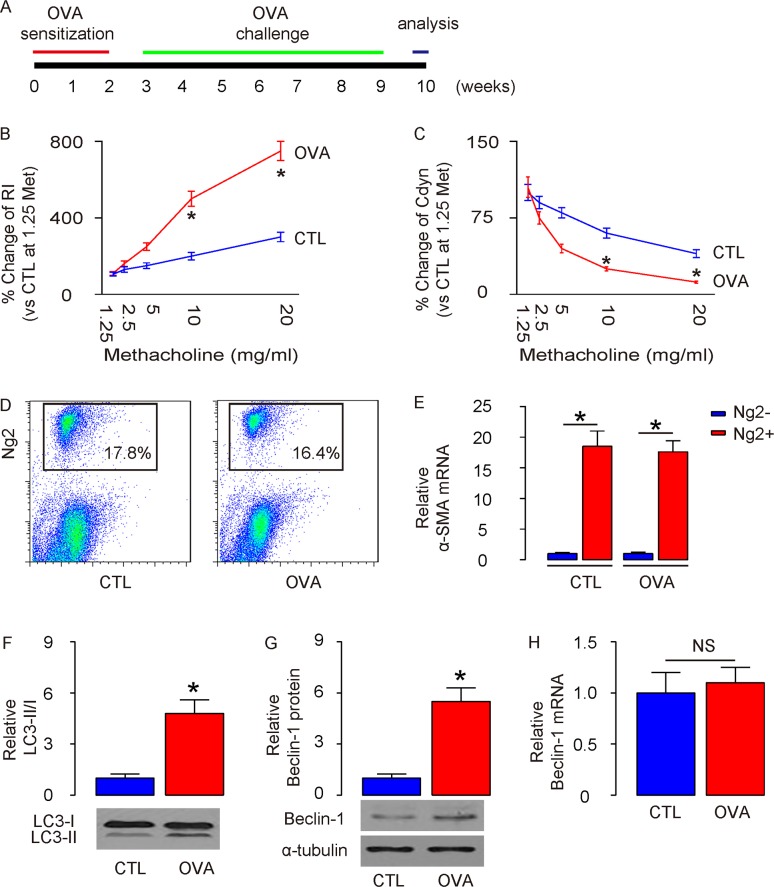
Enhanced ASM cell autophagy is detected in mouse lung after OVA treatment **(A)** Schematic of OVA model. Mice were first sensitized to alum-adsorbed OVA for 2 weeks, and then exposed to repeated airway provocation for another 7 weeks to establish airway hyper-sensitivity. **(B)** Dose-dependent responses in lung resistance (Rl) to methacholine. **(C)** Dose-dependent dynamic compliance (Cdyn) in response to methacholine. **(D)** Representative flow charts for purification of ASM cells from lung digests at the end of 7-weeks’ OVA challenge, based on Ng2 expression. **(E)** RT-qPCR for α-smooth muscle actin (α-SMA) in Ng2+ and Ng2- cells. **(F)** Western blotting for LC3 in Ng2+ and Ng2- cells. **(G-H)** Western blotting (G) and RT-qPCR (H) for Beclin-1 in Ng2+ and Ng2- cells. *p<0.05. NS: non-significant. N=10.

A mouse allergic asthma model was applied as described before [11]. Briefly, mice were first sensitized to alum-adsorbed OVA for 2 weeks, and then exposed to repeated airway provocation for another 7 weeks to establish airway hyper-sensitivity (Figure 1A). The asthma establishment was validated by a dose-dependent increase in lung resistance (Rl) (Figure 1B) and a dose-dependent decrease in dynamic compliance (Cdyn) in response to methacholine, a cholinergic stimulus (Figure 1C). Next, we purified ASM cells from lung digests at the end of 7-weeks’ OVA challenge, based on Ng2 expression, by flow cytometry (Figure 1D). We found that the purified Ng2-postive cells in lung digests from either control (CTL) mice or OVA mice were highly enriched for α-smooth muscle actin (α-SMA), a specific marker for ASM cells in lung (Figure 1E). We thus examined the autophagy levels in these purified ASM cells. Significant higher LC3-II/I ratio was detected in ASM cells from OVA-treated mice, compared to CTL mice (Figure 1F), likely resulting from augmentation of the protein levels of autophagy-regulator Beclin-1 (Figure 1G). However, unlike protein, the mRNA levels of Beclin-1 did not alter in ASM cells from OVA-mice (Figure 1H). Thus, the transcription of Beclin-1 in ASM cells remained unchanged but the Belcin-1 protein increased to enhance autophagy, which suggests presence of post-transcriptional control for Beclin-1 after OVA treatment.

### Enhanced autophagy in ASM cells from OVA-treated mice likely results from loss of suppression of protein translation of Beclin-1 by miR-384

**Figure 2:**
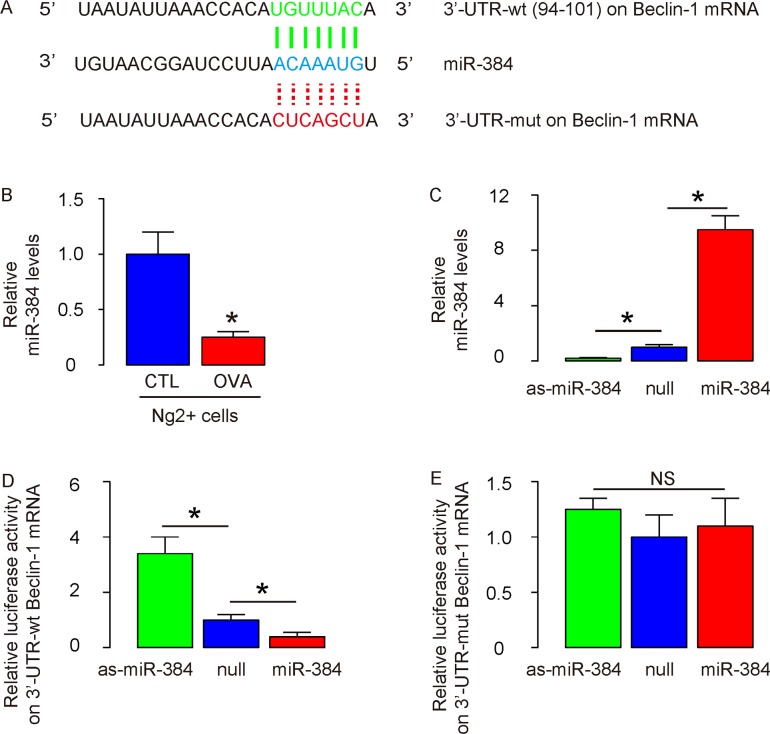
Enhanced autophagy in ASM cells from OVA-treated mice likely results from loss of suppression of protein translation of Beclin-1 by miR-384 **(A)** Bioinformatics to illustrate the binding site for miR-384 on wildtype (wt) 3’-UTR of Beclin-1 mRNA as well as the mutate (mut) 3’-UTR for luciferase reporter assay. **(B)** RT-qPCR for miR-384 in ASM cells after OVA. **(C)** RT-qPCR for miR-384 in ASM cells transfected with miR-384 mimics, antisense for miR-384 (as-miR-384) or null controls (null). **(D-E)** luciferase reporters containing the 3’-UTR of Beclin-1 mRNA (D) or a mutated 3’-UTR of Beclin-1 mRNA in the miR-384 binding site (E) were constructed. These reporter plasmids were co-transfected with miR-384 mimics, as-miR-384 or null controls to ASM cells and luciferase activity was determined. *p<0.05. NS: non-significant. N=10.

Hence, we examined whether Beclin-1 protein translation might be regulated by miRNA-mediated suppression. Bioinformatics were thus performed, showing that miR-384 is such a miRNA that targets 3’-UTR of Beclin-1 mRNA at one binding site (Figure 2A), and decreased expression level in ASM cells after OVA (Figure 2B). The expression levels of miR-384 in ASM cells was altered by transfection with miR-384 mimics, antisense for miR-384 (as-miR-384) or null controls (null), shown by RT-qPCR (Figure 2C). Next, luciferase reporters containing the 3’-UTR of Beclin-1 mRNA or a mutated 3’-UTR of Beclin-1 mRNA in the miR-384 binding site were constructed (Figure 2A). We found that miR-384 markedly inhibited the luciferase activity of the vector containing the wild-type binding site, whereas the as-miR-384 increased the luciferase activity in ASM cells (Figure 2D). On the other hand, transfection of either miR-384 or as-miR-384 did not affect the luciferase activity of the reporter for 3’-UTR of Beclin-1 mRNA carrying the mutated miR-384 binding site in ASM cells (Figure 2E). Together, these data suggest that enhanced autophagy in ASM cells from OVA-treated mice may result from loss of suppression of protein translation of Beclin-1 by miR-384.

### Generation of specific ASM-expressing viruses

**Figure 3:**
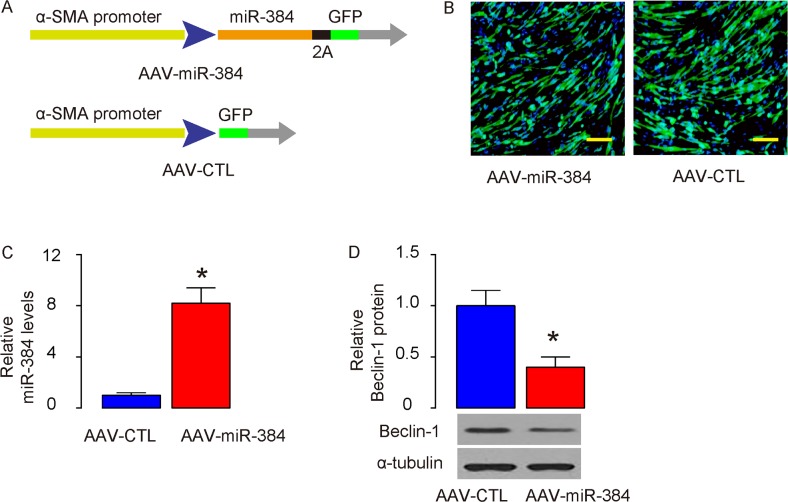
Generation of specific ASM-expressing viruses **(A)** Schematic of generation of adeno-associated viruses (AAVs) carrying miR-384 (AAV-miR-384) or null (AAV-CTL) under control of an α-SMA promoter. **(B)** Visualization of the transduced ASM cells in culture by GFP. **(C-D)** The transduced cultured ASM cells were examined for miR-384 by RT-qPCR (C), and Beclin-1 by Western blotting (D). *p<0.05. N=5. Scale bars are 20 μm.

In order to figure out the effects of the suppressed autophagy of ASMs on asthma development or airway hypersensitivity, we generated Adeno-associated viruses (AAVs) carrying miR-384 (AAV-miR-384) or null (AAV-CTL) under control of an α-SMA promoter, to specifically express the transgene in ASM cells (Figure 3A). These viruses also carried a GFP reporter to allow visualization the transduced cells (Figure 3B). The transduced cultured ASM cells were examined for miR-384 by RT-qPCR (Figure 3C), and Beclin-1 by Western blotting (Figure 3D), which confirmed the overexpression of miR-384 and suppression of Beclin-1 protein in these cells.

### Successful *in vivo* re-expression of miR-384 in ASM cells

**Figure 4:**
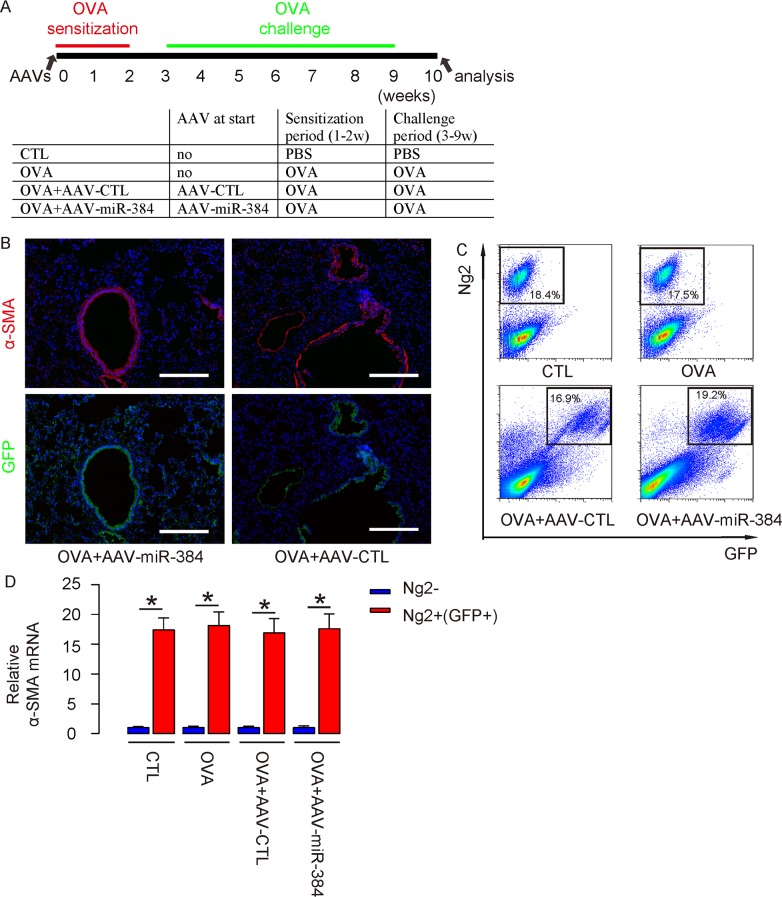
Successful *in vivo* re-expression of miR-384 in ASM cells **(A)** Schematic of the experiment: AAVs were used to treat mice at the beginning of OVA sensitization. Four group of mice of 10 of each were included in this experiment. Group 1, the mice received saline only as control for OVA (CTL). Group 2, mice received OVA treatment only (OVA). Group 3, mice received OVA and intranasal injection of AAV-CTL (OVA+AAV-CTL). Group 4, mice received OVA and intranasal injection of AAV-miR-384 (OVA+AAV-miR-384). **(B)** Immunostaining for α-SMA and GFP in AAVs/OVA-treated mice. Nuclei were stained with DAPI. **(C)** (Transduced) ASM cells were thus isolated from 4 groups, shown by representative flow charts. **(D)** RT-qPCR for α-smooth muscle actin (α-SMA) in Ng2+(GFP+) and Ng2- cells. *p<0.05. NS: non-significant. N=10. Scale bars are 100 μm.

Then, we used these AAVs to treat OVA mice. Four group of mice of 10 of each were included in this experiment. Group 1, the mice received PBS only as control for OVA (CTL). Group 2, mice received OVA treatment only (OVA). Group 3, mice received OVA and intranasal injection of AAV-CTL (OVA+AAV-CTL). Group 4, mice received OVA and intranasal injection of AAV-miR-384 (OVA+AAV-miR-384) (Figure 4A). At analysis, we detected exclusive expression of GFP on α-SMA-positive ASM cells (Figure 4B). (Transduced) ASM cells were thus isolated from 4 groups by flow cytometry (Figure 4C). We found that the purified ASM cells in lung digests from either groups were highly enriched for α-SMA (Figure 4D).

### Overexpression of miR-384 in ASM cells significantly reduces ASM cell autophagy and attenuates OVA-induced airway hypersensitivity

**Figure 5:**
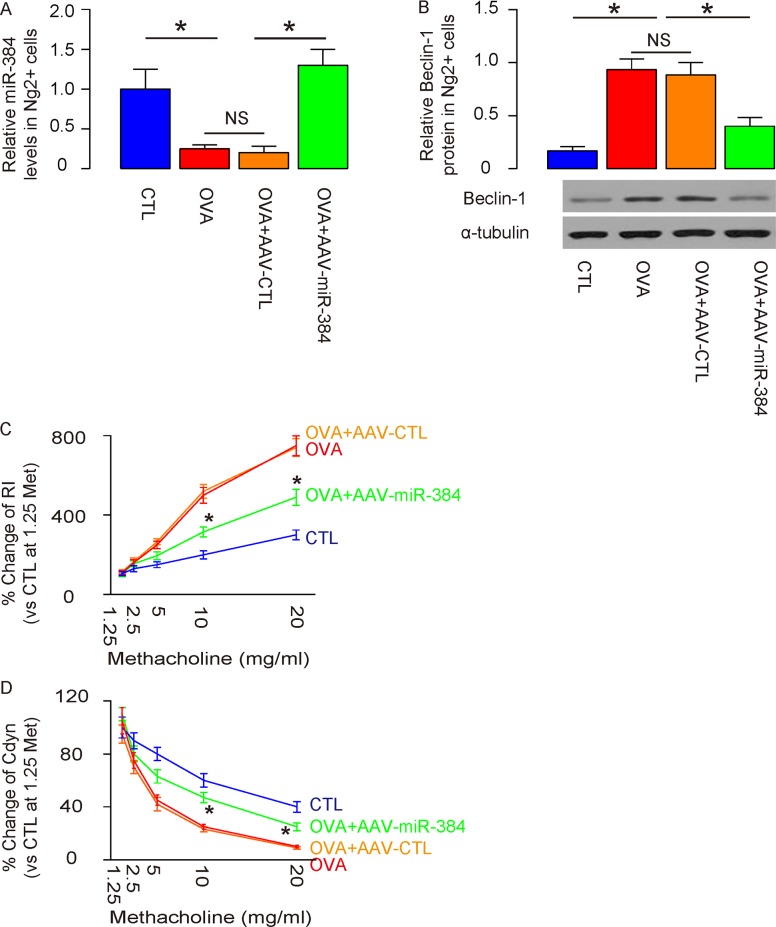
Overexpression of miR-384 in ASM cells significantly reduces ASM cell autophagy and attenuates OVA-induced airway hypersensitivity **(A)** RT-qPCR for miR-384 in purified ASM cells from 4 groups. **(B)** Western blotting for Beclin-1 in purified ASM cells from 4 groups. **(C)** Dose-dependent responses in lung resistance (Rl) to methacholine. **(D)** Dose-dependent dynamic compliance (Cdyn) in response to methacholine. *p<0.05. In C and D, statistics were performed to compare group OVA+AAV-CTL and OVA+AAV-miR-384. NS: non-significant. N=10.

Overexpression of miR-384 in ASM cells by AAV-miR-384 transduction was confirmed by RT-qPCR in purified ASM cells (Figure 5A), resulting in abolishment of increases in Beclin-1 protein levels by Western blotting (Figure 5B). Moreover, overexpression of miR-384 in ASM cells by AAV-miR-384 significantly reduced the OVA-induced dose-dependent increase in RI (Figure 5C) and significantly attenuated the OVA-induced dose-dependent decrease in Cdyn in response to methacholine (Figure 5D). These data demonstrate that re-expression of miR-384 in ASM cells significantly reduces ASM cell autophagy and attenuates OVA-induced airway hypersensitivity.

## DISCUSSION

Asthma is a chronic respiratory disease afflicting 200 million people worldwide including a great percentage of children [1, 2]. Asthma manifests many symptoms including wheezing, breathlessness and chest tightness, and interacts with other diseases like sinusitis, obstructive sleep apnea and cardiac dysfunction [1, 2].

ASM cells are key players in airway disorder, augmented inflammation, narrowing and remodeling. Increased ASM cell mass has been suggested to contribute to all asthma-associated features, and is traditionally believed to result from increased proliferation and reduced apoptosis [3]. However, recent studies on cell biology revealed that autophagy, as a highly conserved catabolic process in which misfolded or unnecessary proteins and damaged organelles are delivered to lysosomes for degradation and recycling, may contribute to alteration of cell mass *in vivo* [12]. However, whether autophagic status of ASM cells in the asthma setting may be altered is unknown [13]. Hence, we addressed this question here.

One typical hallmark of autophagy is the formation of double-membrane autophagosomes, which fuse with lysosomes to form autophagolysosomes [14]. LC3 is a protein that targets to the autophagosomal membranes. LC3 has 2 forms: LC3-I (18 kDa) and LC3-II (16 kDa). Newly synthesized LC3 are cleaved immediately to produce cytosolic LC3-I. LC3-I undergoes a series of ubiquitination-like modifications to generate tightly membrane-bound protein LC3-II which is potently attached to the preautophagosomal structure and autophagosomes. The relative amount of membrane-bound LC3-II reflects the abundance of autophagosomes, as an exclusive marker for autophagy [14]. Therefore, we used ratio of LC3-II vs LC3-I to evaluate autophagy levels of the cells.

Ng2 is a specific marker for ASM cells [15]. Although α-SMA is a more commonly used marker for ASM cells, it is a cytosolic protein which could not be used as a surface marker for isolation of ASM cells [15]. Thus, we used α-SMA to prove the purity of Ng2-positive cells as ASM cells, but used α-SMA promoter to drive the transgene to express it specifically in ASM cells. The specificity of these viruses were further confirmed in the flow cytometry based analysis and sorting. While in CTL and OVA conditions, no GFP+ cells were detected in the lung digests, suggesting that the GFP+ cells in the other 2 conditions with AAVs were not autofluorescent cells. In OVA+AAV-CTL and OVA+AAV-miR-384 conditions, most Ng2+ cells are GFP+ cells, confirming the specific transduction of ASM cells, and the high transduction efficiency of the system.

We applied both OVA and OVA+AAV-CTL as controls in *in vivo* studies, and got similar results. These data suggest that the viral infection itself, although may have an effect on immunity, has little effects on the conditions that we examined here, and should not affect our interpretation of the data [16].

Here, our work highlights a critical role of miRNAs in the regulation of pathogenesis of asthma. The exact mechanism that leads to the alteration of miR-384 levels in ASM cells after OVA is still unknown and should be addressed in future studies. To summarize, here we showed evidence for miR-384 as a novel target to experimentally control the development of asthma and airway hypersensitivity.

## MATERIALS AND METHODS

### Protocols and animals

All mouse experiments were approved by research committee of Zhengzhou University. Male C57BL/6 mice of 10 weeks of age (Experimental Animal Centre of Zhengzhou University, Zhengzhou, China) and kept in a specific pathogen-free environment for the duration of the current study.

### Experimental animal models

Male C57BL/6 mice of 10 weeks of age housed in a specific Pathogen-free condition were sensitized with an intraperitoneal injection of 50 μg OVA (OVA, grade V; Sigma-Aldrich, St. Louis, MO, USA) with 2mg aluminum hydroxide gel (Alum; Sigma-Aldrich) at a frequency of once per week from week 0 to week 2. Mice were then challenged with 50 μg OVA by intranasal administration 3 times per week from week 3 to week 9. Mice that received PBS of same volume and frequency were used as control (CTL). For AAVs injection, mice received intranasal injection of 3×10^11^ AAVs at the beginning of the study (week 0).

### Airway hyper-responsiveness

Airway hyper-responsiveness was determined using restrained invasive plethysmography assay. Briefly, after the mice were anesthetized, the trachea was exposed to allow a cannula that connects an inline nebulizer and ventilator to be inserted. Mice were then challenged with aerosolized PBS followed by increasing doses of methacholine (Sigma-Aldrich). Airway resistance (AR) and dynamic compliance (Cdyn) were determined by analysis of pressure and flow waveforms.

### Flow cytometry

Mouse lung was dissociated into single cells by incubation with 0.015% Trypsin at 37°C for 40-50 minutes. The single cell fraction was incubated with Alexa Fluor 647-conjugated Ng2 antibody (Becton-Dickinson Biosciences, San Jose, CA, USA) for 15 minutes before subjected to florescence-activated cell sorting. GFP was detected by direct fluorescence.

### Bioinformatics and dual luciferase-reporter assay

MiRNAs targets were predicted using the algorithms TargetSan [17]. Luciferase reporter assay was done using a Luciferase Reporter Gene Detection Kit (Sigma-Aldrich), as instructed. MiR-384 mimics, antisense for miR-384 (as-miR-384) or null controls (null represents empty sequence for the miR-384 insert) were purchase from RiboBio Co., Ltd. (Guangzhou, Guangdong, China). Beclin-1 mRNA 3'-UTR wildtype clone (wt) and Beclin-1 mRNA 3'-UTR with a site mutation at the miR-384 binding site (mut) were purchased from RiboBio Co., Ltd.

### Plasmid and AAV production

The miR-384 mimics, or null control plasmids were cloned into a pAAV-CAG-GFP plasmid (Clontech, Mountain View, CA, USA), the CAG promoter of which was replaced by an α-SMA promoter cloned from mouse genomic DNA. To generate AAVs, HEK293T cells were co-transfected with 10 μg of the prepared plasmids (pAAV-α-SMAp-miR-384-GFP or pAAV-α-SMAp-GFP) and 5 μg each of packaging plasmids using Lipofectamine-2000 (Invitrogen, CA, Carlsbad, USA). The viruses were purified using CsCl density centrifugation and the titrated with a quantitative densitometric dot-blot assay.

### RT-qPCR

Total RNA was extracted from purified ASM cells by column method using the RNeasy Mini kit (Qiagen, Valencia, CA, USA) according to manufacturer's instructions and quantified with the Nanodrop system (Thermo Scientific, Waltham, MA, USA). cDNA was obtained from the extracted RNA by reverse transcription with the RT2 First Strand Kit (Qiagen) as instructed. The cDNA was then subjected to RT2 SYBRgreen qPCR and analyzed using the 2-ΔΔCt method. All primers were purchased from Qiagen. Values of gene of interest were first normalized against housekeeping gene and then compared to the experimental control.

### Western blotting

Cell lysates were obtained with the M-Per Protein Extraction Reagent (Thermo Scientific, Rockford, IL, USA) as instructed and protein concentration was determined with BCA assay (Thermo Scientific). Primary antibodies for Western Blot are anti-LC3 (1:500), anti-Beclin-1 (1:500) and anti-α-tubulin (1:1000; all from Cell Signaling, San Jose, CA, USA). Secondary antibody is HRP-conjugated anti-rabbit (1:2000; Jackson ImmunoResearch Labs, West Grove, PA, USA). Western blot quantification was performed using NIH ImageJ software (Bethesda, MA, USA).

### Immunohistochemistry

Mouse lung dissected out and fixed in 4% paraformaldehyde for 4 hours, cyro-protected in 30% sucrose overnight, and then sectioned in 7 μM. Primary antibody is rabbit polyclonal anti-α-SMA (R&D System, Los Angeles, CA, USA). Secondary antibody is Alexa Fluor 568-conjugated anti-rabbit (Jackson ImmunoResearch Labs). 4',6-diamidino-2-phenylindole (DAPI) was used to stain nuclei at the end of the staining.

### Statistical analyses

Data were analyzed using one-way ANOVA with a Bonferroni correction, followed by Fisher’ Exact Test for comparison of two groups (GraphPad Prism, GraphPad Software, Inc. La Jolla, CA, USA), shown as the mean ± S.D. The p value less than 0.05 was considered significant.
